# Serum Levels of IL-33 and Correlation with IL-4, IL-17A, and Hypergammaglobulinemia in Patients with Autoimmune Hepatitis

**DOI:** 10.1155/2018/7964654

**Published:** 2018-06-24

**Authors:** Ma Liang, Zhang Liwen, Zhuang Yun, Ding Yanbo, Chen Jianping

**Affiliations:** ^1^Department of Digestive Disease, The First People's Hospital of Changzhou, The Third Affiliated Hospital of Soochow University, Changzhou, Jiangsu, China; ^2^Department of Pediatrics, The Second People's Hospital of Changzhou, Affiliated Hospital of Nanjing Medical University, Changzhou, Jiangsu, China

## Abstract

This study investigated the role of IL-33 in the pathogenesis of autoimmune hepatitis (AIH). The levels of IL-33/sST2 and Th1/Th2/Th17-type cytokines were determined by enzyme-linked immunosorbent assay in serum samples obtained from 30 AIH patients and 20 healthy controls (HCs). In addition, a murine model of experimental AIH (EAIH) was established to investigate the role of IL-33 in disease progression. The serum levels of IL-33, sST2, Th17 cytokines (IL-17A), Th1 cytokines (IFN-*γ*, TNF-*α*), and Th2 cytokines (IL-4) were significantly elevated in AIH patients compared to HCs. Following immunosuppression therapy, serum levels of IL-33 and sST2 were significantly decreased. Additionally, the serum levels of IL-33 in AIH patients were correlated positively with markers of hypergammaglobulinemia (IgG, IgM, and IgA) and liver injury (*γ*-GT/ALP). Also, the serum levels of IL-33 in AIH patients were correlated positively with proinflammatory cytokine levels (IL-17A and IL-4). Interestingly, treatment of EAIH mice with a specific IL-33 neutralizing antibody significantly reversed the increasing trend in serum ALT/AST and inhibited the production of the type 2 (IL-4) and type 17 cytokines (IL-17) but not the type 1 cytokine (IFN-*γ*). Our findings highlight the possible role of the IL-33/sST2 axis in the progression of AIH, opening a new door for developing a novel therapeutic strategy for AIH.

## 1. Introduction

Autoimmune hepatitis (AIH) is a progressive autoimmune inflammatory disease that is characterized by elevated serum levels of liver enzymes, hypergammaglobulinemia, autoantibodies, and hepatic inflammatory infiltrates [1]. Based on the nature of autoantibodies, AIH is classified as type 1 or type 2. Type I (AIH-1) is the most observed type of AIH and is positive for antinuclear antibodies (ANAs) and/or anti-smooth muscle antibodies (ASMAs), whereas type 2 (AIH-2) is positive for anti-liver kidney microsome 1 (ALKM 1). The etiology of AIH is unknown; however, both immunogenetic background and environmental parameters are implicated, such as human leucocyte antigen (HLA)-DR3 and HLA-DR4 [2]. Immune responses against self-liver antigens that are not properly controlled by impaired regulatory T cells are believed to be implicated in AIH-induced liver injury. Growing evidence has shown that activation of follicular helper T cells (TFH) and imbalance of Th1/Th2 cells are closely associated with the etiology and pathogenesis of AIH [3, 4]. Recent studies also suggested that Th17 cells and their cytokine IL-17 may induce accumulation of various inflammatory cells [5], which contribute to AIH pathogenesis as well. Although the role of T cell-mediated immune inflammation in AIH has been elucidated in several in vitro and in vivo studies [3–7], its upstream regulatory process is not fully understood. As a first-line of therapeutic intervention, AIH patients are treated with immunosuppressive and/or prednisolone therapy. However, the efficacy of this treatment is far from satisfactory in a substantial proportion of AIH patient [8]. Therefore, a better understanding of the immunopathogenesis of AIH and the development of new therapeutic strategies will be of great importance in the management of the disease.

Interleukin- (IL-) 33 is a newly described cytokine belonging to the IL-1 super family of cytokines. IL-33 is a ligand for the IL-1 receptor-related protein ST2 (IL1RL1/ST2) and IL-1 receptor accessory protein (IL-1RaP) receptors. Interaction of IL-33 with these receptors triggers the MyD88 and nuclear factor- (NF-) *κ*B-related signal pathway [9]. ST2 has three protein isoforms: a soluble form (sST2), a variant form ST2, and membrane-bound form (ST2L) that is expressed on Th2 and mast cells [9, 10]. It is known that IL-33 functions as an “alarmin,” activating Th2 and mast cells through interaction with IL1RL1/ST2 receptor, which attracts a variety of cytokines, including IL-4, IL-6, and IL-10 [11]. Further investigations indicated that interaction between IL-33 and IL1RL1/ST2 receptor not only regulates the Th2 response but also acts as an important component of Th1/Th17-mediated responses and innate immunity-induced inflammation [12, 13]. In addition, the IL-33/ST2 axis appears to play a pivotal role in Th2-driven chronic inflammatory diseases, such as asthma, inflammatory bowel disease, and allergic rhinitis [14–16]. Likewise, in the field of hepatology, IL-33 has been reported to correlate with liver injury in patients with primary biliary cirrhosis (PBC) [17]. Notably, serum levels of IL-33 were closely associated with the degree of liver injury in patients with hepatitis B virus infection [18]. On the other hand, it is still unclear on whether the IL-33/ST2 axis participates in the pathogenesis of AIH. To address this issue in the present study, we examined the serum levels of IL-33 and sST2 in patients with AIH. Furthermore, the influence of IL-33 on the levels of Th1/Th2/Th17 inflammatory cytokines and liver injury was also assessed.

## 2. Material and Methods

### 2.1. Participants

A total of 30 patients with a diagnosis of AIH were recruited sequentially at the inpatient clinic of the Department of Gastroenterology, the First People's Hospital of Changzhou, Third Affiliated Hospital of Soochow University, China. All patients were examined in an active disease state, as defined by an alanine aminotransferase (ALT) value or aspartate aminotransferase (AST) value >50 U/ml. Among the AIH patients, 21 patients were treated with prednisolone alone at a median dose of 100 mg at the time of acute presentation, while the remaining 9 patients received prednisolone in combination with azathioprine at a median dose of 100 mg. As a control group, 20 age-, gender-, and ethnicity-matched healthy controls (HCs) were also recruited from the Department of Medical Examination Center of the First People's Hospital of Changzhou during the same period. Participants in the control group had no history of any chronic inflammatory disease. AIH was diagnosed according to the international criteria for the definitive diagnosis of AIH-1 [19]. The exclusion criteria included any other autoimmune disease, connective tissue disease (CTD), chronic inflammatory disease, a history of viral hepatitis infection, or treatment with immunosuppressive therapies or glucocorticoid therapies within the previous 6 months. All participants signed an informed consent form prior to the initiation of the study, and the study was approved by the Ethical Committee of the First People's Hospital of Changzhou.

### 2.2. Animals

Adult C57BL/6 female mice were purchased from Nanjing Medical University (Jiangsu, China) and housed at a controlled temperature with light-dark cycles and free access to food and water. The experiments had been approved by the animal experimentation committee, and guidelines for humane care for laboratory animals were observed. Each experiment was carried out on five animals per group and repeated at least three times.

### 2.3. Induction of Experimental Autoimmune Hepatitis (EAIH)

Liver antigens were always prepared freshly as described previously from C57BL/6 female mice after perfusion of livers with phosphate-buffered saline (PBS). Livers were homogenized on ice, and nuclei and remaining intact cells were centrifuged at 150g for 10 minutes. Subsequently, the supernatants were centrifuged for 1 h at 100,000g, and the remaining supernatants were used for immunization (called S100). Induction of experimental autoimmune hepatitis (EAIH) was achieved by intraperitoneal injection of the mice with freshly prepared S-100 antigen at a dose of 0.5–2 mg/mL in 0.5 mL PBS that had been emulsified in an equal volume of complete Freund's adjuvant (CFA) on day 0. A booster dose was given on day 7 as well [20]. Disease severity was assessed histologically on day 28 when the peak of disease activity was observed. Disease severity was graded on a scale of 0 to 3 by a researcher who was blinded to the sample identity.

### 2.4. Histological Evaluation

Liver tissues from sacrificed animals were fixed with 4% (*v*/*v*) paraformaldehyde, dehydrated through a graded series of sucrose, frozen in optimal cutting temperature (OCT) compound (Tissue TCK, Miles Elkhart, IN, USA), and stored at −80°C. Then, 5 *μ*m cryostat sections were stained by hematoxylin and eosin (HE) for estimation of the degree of inflammatory cell infiltration. The liver histology of EAIH was scored under light microscopy according to a modified Scheuer scoring scale, with scores assigned for lobular inflammation (0, none; 1, mild-scattered foci of lobular-infiltrating lymphocytes; 2, moderate numerous foci of lobular-infiltrating lymphocytes; 3, severe/extensive panlobular-infiltrating lymphocytes).

### 2.5. Effects of Anti-m (Mouse)IL-33 Abs on Hepatitis Function in EAIH Mice

A total of 10 EAIH mice were randomly divided into two groups (5 mice/group): a control group and a test group. The test group was injected with a single dose of 4 *μ*g anti-m (mouse)IL-33 antibodies (eBioscience, San Diego, CA, USA), dissolved in pyrogen-free saline solution, intravenously via the tail vein. All mice were sacrificed on day 7, and then, the blood and liver samples were collected. The blood serum was separated for measurement of liver enzymes and proinflammatory cytokines.

### 2.6. Measurement of IL-33 and sST2 by ELISA

The concentrations of IL-33 and sST2 were determined by enzyme-linked immunosorbent assay (ELISA) in the serum samples of human participants and laboratory animals using human and mice IL-33/Sst2 ELISA kits, respectively, according to the manufacturer's instruction (Roche Diagnostics, Lewes, UK). Briefly, the detection ranges of the IL-33 and sST2 ELISA kits were 0–16 ng/L and 0–1.6 ng/L, respectively.

### 2.7. Cytometric Bead Arrays for Serum Cytokines

The serum levels of Th1 cytokines (tumor necrosis factor- (TNF-) *α*, interferon- (IFN-) *γ*), Th2 cytokines (IL-4, IL-6), and a Th17 cytokine (IL-17A) were also determined in the sera of AIH patients and HCs by cytometric bead array (CBA), according to the manufacturer's protocol (BD Biosciences, San Jose, CA, USA). The serum concentrations of cytokines were quantified using the CellQuest Pro and CBA software (Becton Dickinson) on a FACSCalibur cytometer (BD Biosciences).

### 2.8. Statistical Analysis

The differences between groups were analyzed by Mann–Whitney test for unpaired comparison or Wilcoxon signed-rank test for paired comparison. The correlation between different variables was evaluated using the Spearman's rank correlation test. A *P* value < 0.05 was considered statistically significant. All statistical analyses were performed using SPSS 18.0 software (SPSS Inc., Chicago, IL, USA).

## 3. Results

### 3.1. Demographic and Clinical Characteristics of Study Population

The baseline (before treatment) demographic characteristics of the AIH patients and HCs are summarized in Table 1. The levels of serum liver enzymes (ALT, AST, *γ*-GT, and ALP) and white blood cell (WBC) count were significantly higher in AIH patients than those in HCs. Furthermore, 23 of 30 AIH patients tested positive for anti-ANAs, while two tested positive for anti-SMA antibodies. In addition, the levels of serum IgG, IgM, and IgA were also significantly higher in AIH patients than those in HCs. These results indicate the presence of liver injury and hypergammaglobulinemia in AIH patients.

### 3.2. Serum Levels of IL-33/sST2 and Th1/Th2/Th17-Related Cytokines in AIH Patients and HCs

**Figure 1:**
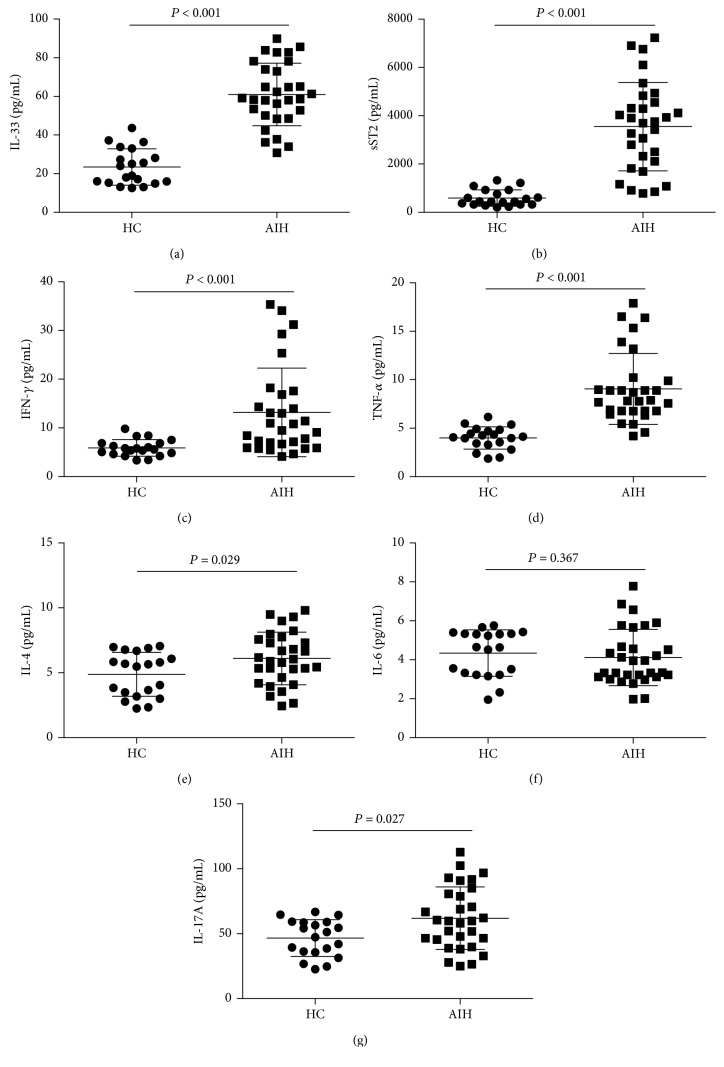
Serum levels of IL-33, sST2, and other cytokines in AIH patients and HCs. Serum levels of (a) IL-33, (b) sST2, (c) IFN-*γ*, (d) TNF-*α*, (e) IL-4, (f) IL-6, and (g) IL-17A in AIH patients during active state and in HCs. Data shown are the mean levels of each serum cytokine in individual subject from two separate experiments. The horizontal lines indicate the mean values for the different groups.

In the acute phase, the serum levels of IL-33 and sST2 in the AIH patients were significantly higher than those in HCs (Figures 1(a) and 1(b), *P* < 0.001). Likewise, the serum levels of IFN-*γ*, TNF-*α*, IL-4, and IL-17A were also elevated in AIH patients compared to those in HCs (Figures 1(c)–1(e) and 1(g), *P* < 0.05). On the other hand, the serum level of IL-6 did not differ significantly between AIH patients and HCs (Figure 1(f)).

### 3.3. Correlations of IL-33 with Other Cytokines in the Serum of AIH Patients

**Figure 2:**
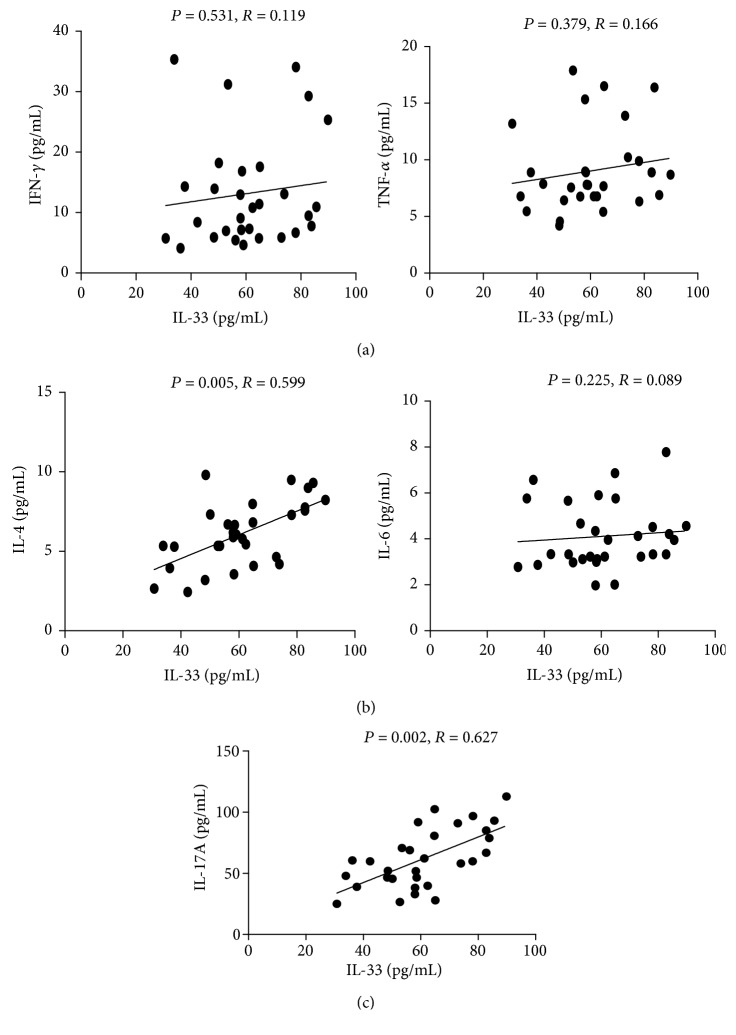
Correlation between IL-33 and other cytokines in AIH patients. (a) Correlation between IL-33 and type 1 cytokine (IFN-*γ*/TNF-*α*) in serum of AIH patients; (b) correlation between IL-33 and type 2 cytokine (IL-4/IL-6) in serum of AIH patients; (c) correlation between IL-33 and type 17 cytokine (IL-17A) in serum of AIH patients.

To the best of our knowledge, the relationships between IL-33 and proinflammatory cytokines in the serum of AIH patients have not yet been investigated. We found significant positive correlations between the serum levels of IL-33 and IL-4 (Th2 cytokine; Figure 2(b), *P* < 0.05) and IL-17A (Th17 cytokine; Figure 2(c), *P* < 0.05) in AIH patients. However, we did not observe any significant correlation between IL-33 and IFN-*γ*, TNF-*α* (Th1 cytokines; Figure 2(a)) and IL-6 levels (Figure 2(b)) in AIH patients.

### 3.4. Correlations of IL-33 with Clinical Measures in AIH Patients

**Figure 3:**
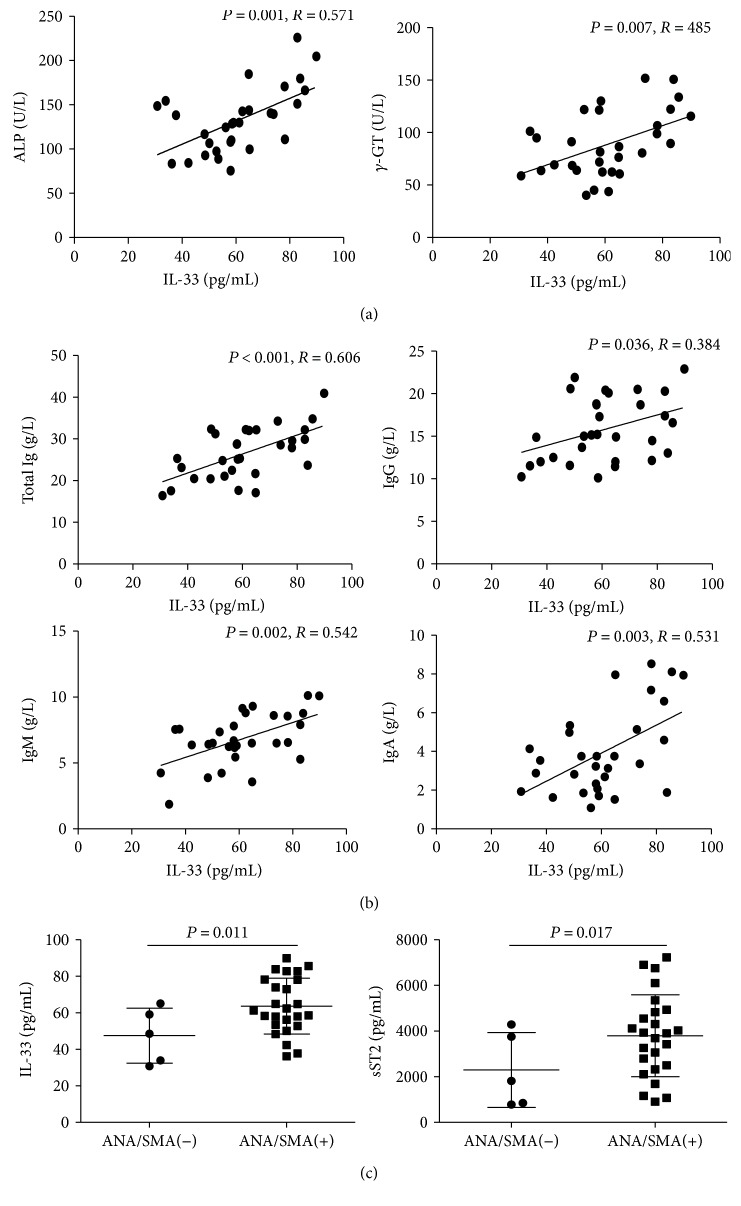
Correlation between serum levels of IL-33 and the values of clinical parameters in AIH patients. Correlation between serum levels of (a) IL-33 and ALP/*γ*-GGT; (b) IL-33 and total Ig (sum of IgA/IgG/IgM titers), IgG, IgM, and IgA in AIH patients; (c) IL-33/sST2 in the seropositive (25 cases) and seronegative (5 cases) anti-ANA/SMA AIH patients. The horizontal lines indicate the mean values for the different groups.

In addition, we analyzed the potential associations of the levels of IL-33 with the values of clinical parameters in AIH patients. We found that IL-33 levels were positively correlated with the levels of alkaline phosphatase (ALP) and gamma-glutamyltransferase (*γ*-GT) (Figure 3(a)). In addition, the serum levels of IL-33 were also correlated positively with the serum titers of total Ig, IgG, IgM, and IgA (Figure 3(b)). On the other hand, there were no significant correlations between the levels of IL-33 and other clinical parameters tested in this population.

### 3.5. Serum Levels of IL-33/sST2 in AIH Patients with Seropositive and Seronegative Pathogenic Autoantibodies

We found that the serum levels of IL-33 and sST2 in the anti-ANA/anti-SMA+ patients were significantly higher than those in the anti-ANA/anti-SMA− patients (Figure 3(c)).

### 3.6. Levels of Serum IL-33 and sST2 in AIH Patients after Treatment

**Figure 4:**
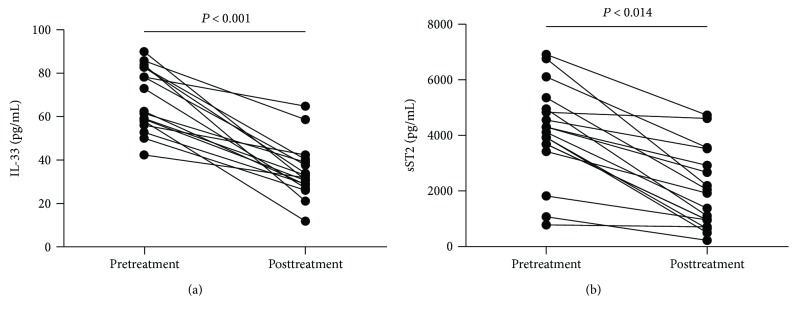
Serum levels of serum IL-33 and sST2 in AIH patients following treatment. (a) Serum levels of IL-33 in 17 AIH patients pre- and posttreatment; (b) serum levels of sST2 in 17 AIH patients pre- and posttreatment. Data shown are the mean levels of each serum cytokine in individual subject from two separate experiments. The horizontal lines indicate the mean values for the different groups.

Furthermore, we analyzed the levels of serum IL-33/sST2 and values of clinical parameters in 17 AIH patients at 8 weeks posttreatment. We found that the serum levels of liver enzymes (ALT, AST, *γ*-GT, and ALP) and the titers of IgG, IgM, and IgA were significantly decreased compared to pretreatments values (Table 2). Similarly, the serum levels of IL-33 and sST2 were significantly decreased compared to the pretreatment levels (Figure 4).

### 3.7. Continuous Time-Dependent Increase in the Levels of Serum IL-33 in EAIH Mice

**Figure 5:**
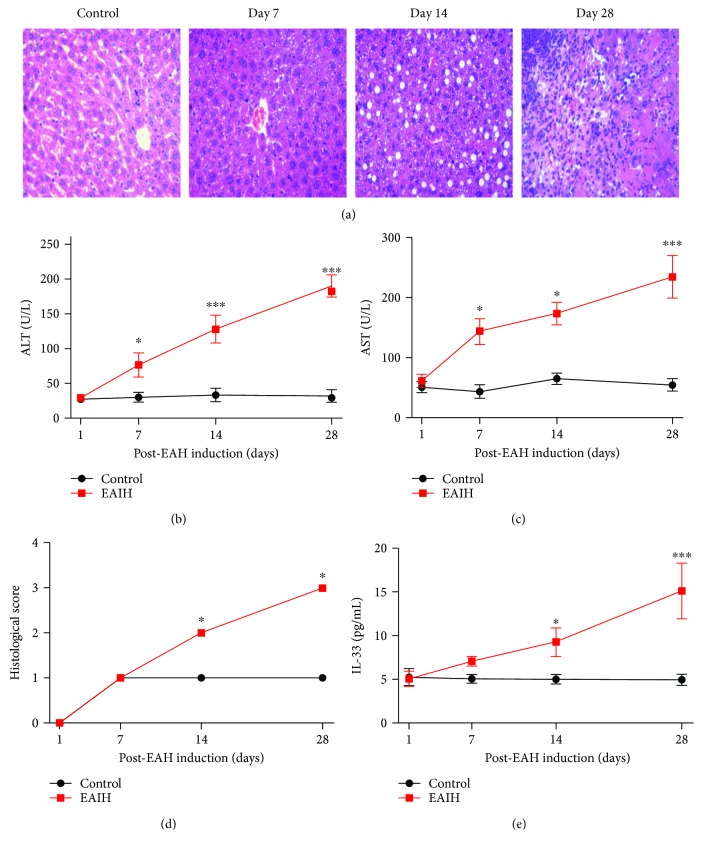
Serum levels of IL-33 in EAIH mice. Ten mice (5 from the EAIH group and 5 from the control group) were killed at each time point (7, 14, and 28 days). (a) Representative histological image of liver lesions in animals after standard induction of EAIH (magnification, 100x). (b) Serum ALT levels were progressively upregulated from 1 to 28 days. (c) Serum AST levels were progressively upregulated from 1 to 28 days. (d) Histological score of liver lesions in mice after standard induction of EAIH. (e) Serum IL-33 levels were progressively upregulated from 1 to 28 days. Data shown are the mean levels of serum IL-33 in each mouse from two separate experiments. The horizontal lines indicate the mean values for the different groups. ^∗^*P* < 0.05; ^∗∗∗^*P* < 0.001.

To better understand the influence of IL-33 in AIH pathogenesis, we successfully established a murine model of EAIH. Compared with the control mice, EAIH mice had obvious liver injury evidenced by liver edema with a rising liver index and elevated serum levels of AST and ALT (Figures 5(a)–5(c)). Notably, the serum level of IL-33 in EAIH mice showed a time-dependent increasing trend compared to that in the control group (Figure 5(d)).

### 3.8. Improvement of Liver Functions in EAIH Mice after Treatment with Anti-mIL-33 Antibodies

**Figure 6:**
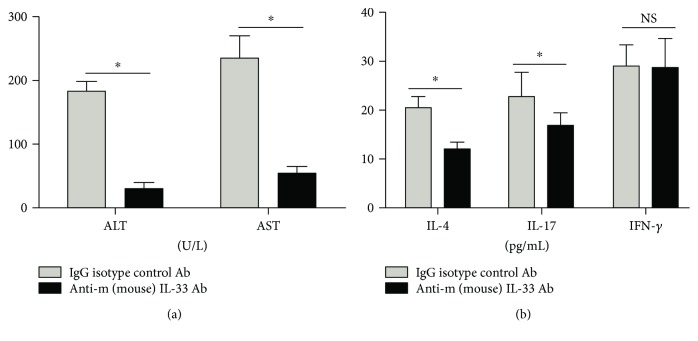
Levels of liver function and Th1/Th2/Th17-type cytokines after treatment with anti-m (mouse)IL-33 antibodies. Ten mice (5 EAIH mice treated with IgG antibodies and 5 EAIH mice treated with neutralizing IL-33 antibodies) were sacrificed on day 7. (a) Serum ALT/AST levels in EAIH mice with/without anti-mIL-33 antibody treatment; (b) serum levels of IFN-*γ*, IL-4, and IL-17 in EAIH mice with/without anti-mIL-33 antibody treatment. Data shown are the mean levels of each serum cytokine in each mouse from two separate experiments. The horizontal lines indicate the mean values for the different groups. ^∗^*P* < 0.05.

We found that the serum levels of AST and ALT were significantly decreased 7 days after treatment with anti-mIL-33 antibodies (Figure 6(a)). Also, serum IL-4 and IL-17A levels were decreased in EAIH mice 7 days after treatment with anti-mIL-33 antibodies (Figure 6(b)). On the other hand, there was no statistically significant difference between the levels of serum IFN-*γ* before and after treatment with anti-mIL-33 antibodies (Figure 6(b)).

## 4. Discussion

IL-33 is a multifunctional cytokine that participates in the pathogenesis of a variety of inflammatory diseases [14–17]. Through the interaction with its receptors (IL1RL1/ST2 and IL-1RaP), IL-33 exerts its biological effects via activating the MAP-kinase and NF-*κ*B signaling pathways [9, 10]. Several studies have demonstrated that dysregulation of ST2/IL-33 signaling can promote the pathogenesis of some Th2-related inflammatory diseases such as asthma and allergic inflammation [14, 15, 21]. Recent studies found a positive correlation between the serum level of IL-33 and the onset of hepatitis B virus- (HBV-) related liver fibrosis, suggesting the possible involvement of IL-33 in the manifestation of chronic hepatitis [22, 23]. Other studies suggest that increased IL-33 levels correlate with the acute-phase inflammatory response in autoimmune diseases such as systemic lupus erythematosus where the levels of serum IL-33 are positively correlated with the erythrocyte sedimentation rate (ESR) and C reactive protein (CRP) [24, 25]. However, several animal studies have also demonstrated that IL-33 treatment can protect against septic shock and reduce inflammation in the lungs of mice infected with influenza virus [26, 27]. Therefore, IL-33 may play dual roles in the immune response, depending on the disease type and the model studied.

Despite these accumulating data, the effects of the IL-33/ST2 axis in patients with AIH are not clear. In this study, we repeated the experiment and found that the levels of serum IL-33 and sST2 were elevated in AIH patients experiencing active-state disease. On the other hand, the treatment with immunosuppressive drugs decreased the levels of serum IL-33 and sST2, suggesting that overexpression of the IL-33/ST2 axis might play an important role in disease progression of AIH. Indeed, the elevated levels of serum IL-33 were positively correlated with liver injury, as indicated by serum GGT and ALP levels. Notably, elevated levels of GGT and ALP are “signature” parameters for AIH [1–3]. Additionally, the levels of serum IL-33 were correlated positively with hyperglobulinemia, which is a hallmark of AIH pathogenesis [28]. Third, we observed dynamic temporal increases in serum expression of IL-33 in EAIH mice along with increases in the levels of ALT/AST and histological scores, which confirm the success of the murine model of EAIH [20, 29]. Lastly and most importantly, specific IL-33-neutralizing antibody significantly reversed this increasing trend in serum ALT/AST in a time-dependent manner in EAIH mice. Taken together, our results suggest that the IL-33/sST2 axis might play an important role in the pathology of AIH. Furthermore, serum IL-33 and sST2 levels could serve as useful predictors of AIH in patients with active-state disease.

Mounting evidence has revealed that various Th-related cytokines contribute to liver inflammation and the autoimmunity process associated with liver tissue damage and repair [11–13]. Proinflammatory Th1 cytokines (IFN-*γ*, TNF-*α*) and the Th17 cytokine (IL-17A) are crucial for Th1 and Th17 cell-mediated immune responses and can promote liver injury by inducing neutrophil infiltration [11–13, 30, 31]. Alternatively, Th2 cytokines (IL-4, IL-6) can regulate B-cell activation and promote the production of ANA and anti-SMA antibodies [32, 33]. In the present study, we further investigated the characteristics of Th1/Th2/Th17-related cytokines in AIH patients. We found that increases in IFN-*γ*, TNF-*α*, and IL-17A were observed in the serum of AIH patients with active-state disease. Although both IL-4 and IL-6 are produced by Th2 cells, our data suggest that the change trend of the IL-4 level was inconsistent with the trend of the IL-6 level. This may be because IL-4 can also be produced by other cell types such as dendritic cells and macrophages [34, 35]. Therefore, these data support the notion that the CD4+ T cells in AIH patients are mainly Th1 and Th17 cells, not Th2 cells. Consistent with the fact that IL-17 and IL-4 are effector factors of the Th17 and Th2 cell responses, we further found that the levels of IL-33 were positively correlated with Th17 cytokines (IL-17A) and the Th2 cytokine (IL-4) in AIH patients. Furthermore, selective anti-mIL-33 antibody inhibited the production of the type 2 cytokine IL-4 and the type 17 cytokine IL-17 but not the type 1 cytokine IFN-*γ*, in EAIH mice. Thus, our results support the previous notion that IL-33 is a critical factor of the functional development of Th2 and Th17 cell responses [11–13, 36]. We are further interested in examining the mechanisms underlying the role of the IL-33/sST2 axis in Th2/Th17 cell activation and differentiation in in vitro and in vivo settings.

In conclusion, our findings indicate that IL-33 is closely associated with the pathogenesis of AIH, and its action might be exerted via Th2/Th17-mediated immune responses.

## Figures and Tables

**Table 1:** Demographic and clinical characteristics of study participants.

Parameters	AIH patients	HCs
Number	30	20
Age (years)	49 (38–76)	51 (41–74)
Gender: female/male	22/8	14/6
ALT (U/L)	126.4 ± 111.9^∗^	27.2 ± 8.2
AST (U/L)	102.4 ± 55.3^∗^	22.7 ± 5.7
*γ*-GT (U/L)	88.9 ± 31.4^∗^	25.1 ± 7.4
ALP (U/L)	132.6 ± 36.8^∗^	89.5 ± 23.6
Anti-ANA(+)	23/30 (76.7%)^∗^	0/20 (0%)
Anti-ANA titer	1 : 640 (1 : 80–1 : 10000)	—
Anti-SMA(+)	2/30 (6.7%)^∗^	0/20 (0%)
Anti-SMA titer	1 : 1000 (1 : 160–1 : 3200)	—
IgG (g/L)	15.8 ± 3.8^∗^	7.8 ± 2.3
IgM (g/L)	6.8 ± 1.9^∗^	2.64 ± 0.87
IgA (g/L)	3.98 ± 2.2^∗^	1.6 ± 1.1
WBC count (^∗^10^9^/L)	7.62 (5.6–11.2)^∗^	5.08 (3.9–9.2)

Data shown are expressed as mean ± SD. Normal values: ALT: <40 IU/L; AST: <40 IU/L; ANA: <1 : 80; SNA: <1 : 80. IgG: normal range = 7–16 g/L; IgM: normal range = 0.7–4.6 g/L; IgA: normal range = 0.4–2.3 g/L. HC: healthy control; AIH: autoimmune hepatitis. ^∗^*P* < 0.05 versus HC.

**Table 2:** Effect of treatment on the values of clinical measures in AIH patients.

Parameters	Before treatment	After treatment
Number	17	17
Age (years)	43 (38–76)	43 (38–76)
Gender: female/male	14/3	14/3
ALT (U/L)	192.3 ± 108.7^∗^	36.8 ± 10.9
AST (U/L)	131.8 ± 55.7^∗^	44.2 ± 11.88
GGT (U/L)	107.1 ± 27.1^∗^	76.1 ± 24.9
ALP (U/L)	153.2 ± 32.7^∗^	95.4 ± 23.7
IgG (g/L)	18.2 ± 2.9^∗^	8.8 ± 3.4
IgM (g/L)	7.7 ± 1.6^∗^	4.52 ± 2.3
IgA (g/L)	3.8 ± 1.5^∗^	2.6 ± 1.0

Data shown are real case number or mean ± SD. Normal values: ALT: <40 IU/L; AST: <40 IU/L. IgG: normal range = 7–16 g/L; IgM: normal range = 0.7–4.6 g/L; IgA: normal range = 0.4–2.3 g/L. HC: healthy control; AIH: autoimmune hepatitis. ^∗^*P* < 0.05 versus posttreatment.

## Data Availability

The data used to support the findings of this study are available from the corresponding author upon request.
